# Do an ecosystem engineer and environmental gradient act independently or in concert to shape juvenile plant communities? Tests with the leaf-cutter ant *Atta laevigata* in a Neotropical savanna

**DOI:** 10.7717/peerj.5612

**Published:** 2018-10-09

**Authors:** Alan N. Costa, Emilio M. Bruna, Heraldo L. Vasconcelos

**Affiliations:** 1 Instituto de Biologia, Universidade Federal de Uberlândia, Uberlandia, Minas Gerais, Brazil; 2 Department of Wildlife Ecology and Conservation, University of Florida, Gainesville, FL, USA; 3 Center for Latin American Studies, University of Florida, Gainesville, FL, USA

**Keywords:** *Atta*, Seedling, Herbivory, Gradient, Ecosystem engineer, Cerrado, Soil fertility, Canopy cover, Tropical, Savanna

## Abstract

**Background:**

Ecosystem engineers are species that transform habitats in ways that influence other species.While the impacts of many engineers have been well described, our understanding of how their impact varies along environmental gradients remains limited. Although disentangling the effects of gradients and engineers on biodiversity is complicated—the gradients themselves can be altered by engineers—doing so is necessary to advance conceptual and mathematical models of ecosystem engineering. We used leaf-cutter ants (*Atta* spp.) to investigate the relative influence of gradients and environmental engineers on the abundance and species richness of woody plants.

**Methods:**

We conducted our research in South America’s *Cerrado*. With a survey of plant recruits along a canopy cover gradient, and data on environmental conditions that influence plant recruitment, we fit statistical models that addressed the following questions: (1) Does *A. laevigata* modify the gradient in canopy cover found in our Cerrado site? (2) Do environmental conditions that influence woody plant establishment in the Cerrado vary with canopy cover or proximity to *A. laevigata* nests? (3) Do *A. laevigata* and canopy cover act independently or in concert to influence recruit abundance and species richness?

**Results:**

We found that environmental conditions previously shown to influence plant establishment in the *Cerrado* varied in concert with canopy cover, but that ants are not modifying the cover gradient or cover over nests. However, ants are modifying other local environmental conditions, and the magnitude and spatial extent of these changes are consistent across the gradient. In contrast to prior studies, we found that ant-related factors (e.g., proximity to nests, ant changes in surface conditions), rather than canopy cover, had the strongest effect on the abundance of plant recruits. However, the diversity of plants was influenced by both the engineer and the canopy cover gradient.

**Discussion:**

* Atta laevigata* in the Cerrado modify local conditions in ways that have strong but spatially restricted consequences for plant communities. We hypothesize that ants indirectly reduce seedling establishment by clearing litter and reducing soil moisture, which leads to seed and seedling desiccation. Altering soil nutrients could also reduce juvenile growth and survivorship; if so these indirect negative effects of engineering could exacerbate their direct effects of harvesting plants. The effects of *Atta* appear restricted to nest mounds, but they could be long-lasting because mounds persist long after a colony has died or migrated. Our results support the hypothesis that leaf-cutter ants play a dominant role in Cerrado plant demography. We suggest the ecological and economic footprint of these engineers may increase dramatically in coming decades due to the transformation of the Cerrado by human activities.

## Introduction

Species that transform habitats or create new ones are known as ecosystem engineers (Jones, Lawton & Shachak, 1994; Jones, Lawton & Shachak, 1997), and they can have major impacts on population dynamics, community composition, and ecosystem function (reviewed in Kleinhesselink, Magnoli & Cushman, 2014; Wright & Jones, 2006). Most research on engineers to date has focused on documenting the magnitude of their impacts on local biodiversity, with more recent work evaluating how these impacts vary spatially (e.g., Badano et al., 2006; Baker et al., 2013; Dibner, Doak & Lombardi, 2015; Kleinhesselink, Magnoli & Cushman, 2014; McAfee, Cole & Bishop, 2016). An emerging area of interest is identifying how the impacts of engineers vary along or even alter environmental gradients (Bertness & Callaway, 1994; Crain & Bertness, 2006), which are ubiquitous and can also exert strong effects on biodiversity (e.g., John et al., 2007). Experimental studies disentangling the effects of engineers and gradients are rare, however, in part because they are challenging to design and implement at the landscape scale. This makes surveys of biodiversity in landscapes where gradients and engineers overlap, coupled with measurements of ecologically relevant environmental parameters, an important tool for advancing conceptual and mathematical models of ecosystem engineering (Hastings et al., 2007; Wright & Jones, 2006).

Brazil’s *Cerrado* is a savanna woodland whose distribution of 2 million km^2^ makes it South America’s second largest biome. Like many other savanna biomes the Cerrado is a mosaic of plant physiognomies ranging from open grassland to forests (Oliveira-Filho & Ratter, 2002). These vegetation types are often found in close proximity (Cardoso et al., 2009), resulting in broad and continuous gradients in canopy cover that can have important implications for local plant biodiversity. Canopy cover in a site is associated with both biotic and abiotic variables that exert strong effects on woody plant recruitment and survivorship (Salazar et al., 2012a); locations with more canopy cover have cooler understories and produce more leaf-litter, which facilitates seedling establishment and enhances survival of recruits by reducing soil water deficits and increasing nutrient availability (Salazar et al., 2012a). In addition, closed-canopy sites also have less cover of the grasses that can inhibit seedling establishment (Hoffmann & Haridasan, 2008).

Also found in the Cerrado is a prominent ecosystem engineer: leaf-cutter ants (*Atta* spp.). They transport tons of soil to the surface as they excavate their massive nests, create mounds whose surface area can reach 100 m^2^ (Alvarado, Berish & Peralta, 1981), harvest copious amounts of plant biomass, farm fungal colonies in chambers up to 10 m below the surface, and alter nutrient cycling and soil properties (reviewed in Farji-Brener & Werenkraut, 2017; Leal, Wirth & Tabarelli, 2014). *Atta* colonies have direct effects on plant populations and communities—they are major seed predators and harvest seedlings to use as the substrate for their fungal gardens (e.g., Costa, Vasconcelos & Bruna, 2017; Silva et al., 2012; Vasconcelos & Cherrett, 1997). In addition to their direct impacts on plants, however, their alteration of soil properties throughout of the landscape may also indirectly influence plant growth, survivorship, or community composition (e.g., Bieber et al., 2011; Garrettson et al., 1998; Meyer et al., 2011; Sosa & Brazeiro, 2012). To date, the potential for engineering by *Atta* to indirectly influence plant communities has primarily been studied in lowland tropical forests (Farji-Brener & Illes, 2000; Leal, Wirth & Tabarelli, 2014). However, the abundance of *Atta* colonies can be 2–3 fold greater in the Cerrado (Costa & Vieira-Neto, 2016), where they have the ability to completely defoliate trees (Mundim et al., 2012). This suggests a novel means by which this ecosystem engineer could indirectly shape plant diversity—by modifying canopy cover gradients, and therefore the local environmental conditions that influence seedling establishment and the subsequent growth of juvenile plants (Correa et al., 2016). The magnitude of these indirect impacts should vary along the gradient, however, because areas where trees are sparse will already be hotter, brighter, and have limited litter on the soil surface.

To elucidate how gradients and ecosystem engineers interact to influence plant biodiversity we used data on the distribution of over 1,200 plants in a Cerrado landscape dominated by the leaf-cutter ant *Atta laevigata*. Our study addressed the following questions: (1) Does *A. laevigata* modify the gradient in canopy cover found in our Cerrado site? (2) Do environmental conditions that influence the establishment of woody plants in the Cerrado vary with canopy cover or proximity to *A. laevigata* nests? (3) Do *A. laevigata* and canopy cover act independently or in concert to influence the abundance and species richness of plant recruits?

## Materials and Methods

### Study site and system

**Figure 1:**
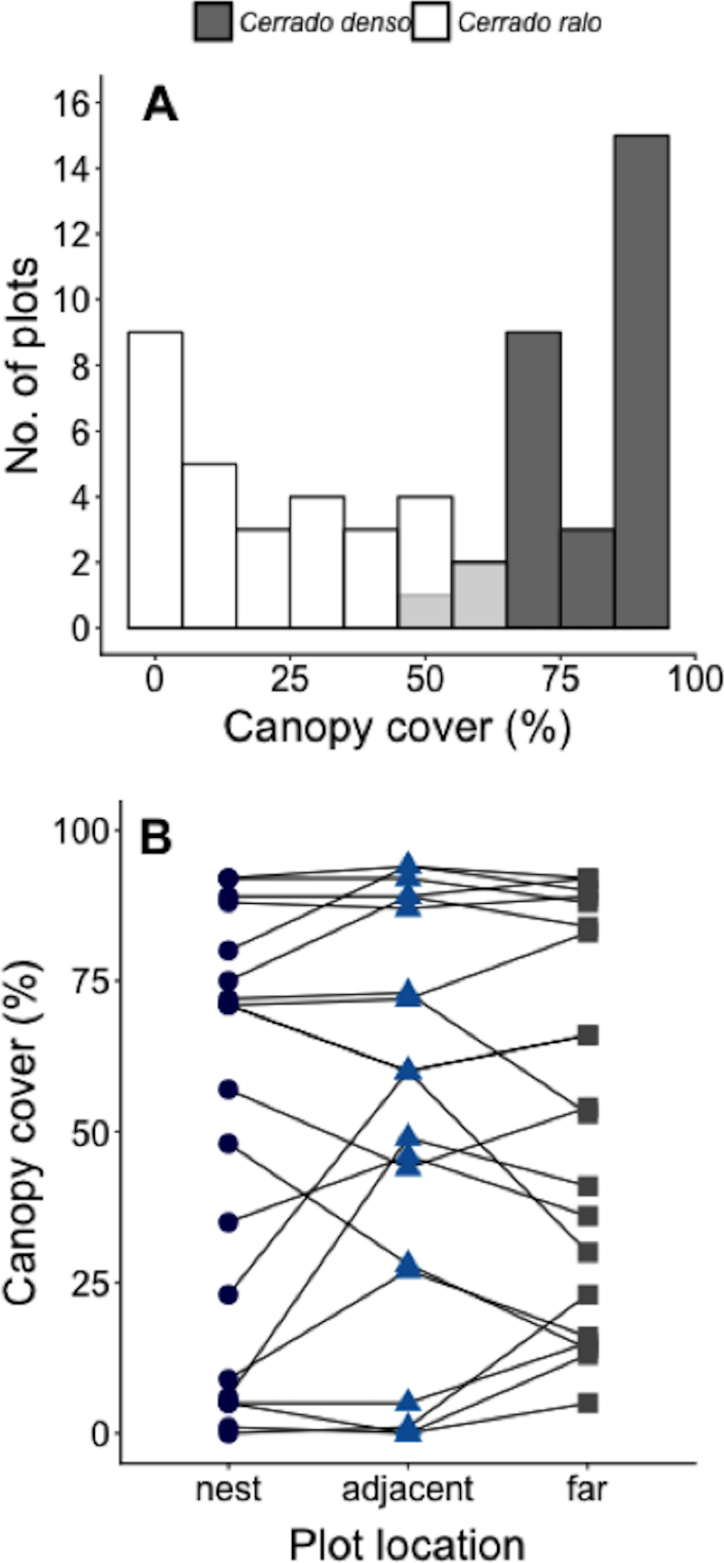
Measurements of canopy cover in our Cerrado site. (A) Number of plots in our *Cerrado* study site with different amounts of canopy cover. Dark gray bars represent plots in the *cerrado denso* vegetation type, while white bars light refer to plots in *cerrado ralo.* Light gray bars indicate overlap in habitat types. Three plots were arranged around each of *N* = 20 leaf-cutter ant (*Atta laevigata*) nests: one in the center of the nest mound, one on the edge of the nest, and one 10 m from the edge of the nest (*N* = 60 plots total). (B) Canopy cover over plots on *Atta laevigata* nests (blue circles), adjacent to nests (blue triangles), and far from nests (gray squares). Canopy cover is independent of plot proximity to the *N* = 20 nests (Table 1), indicating ants are not responsible for or modifying the canopy cover gradient in our study site.

We conducted our study at Panga Ecological Station (19°10′45″S, 48°23′44″W), a 404 ha reserve (Bruna et al., 2010) administered by the Universidade Federal de Uberlândia (UFU). The climate at Panga is highly seasonal, with mean annual temperature of ∼23° and most of the ∼1,600 mm of annual precipitation between October-April (UFU Santa Mônica Climate Station). Most of the major Cerrado vegetation types can be found at Panga Station, including the two known as *cerrado ralo* and *cerrado denso* (Cardoso et al., 2009; Appendix A). *Cerrado ralo* has a dense layer of grasses and herbs and sparsely distributed shrubs and trees typically <3 m tall; the average canopy cover in *cerrado ralo* is ∼30%. *Cerrado denso* has less grass cover and more abundant trees that can reach a height of ca. 6 m; average canopy cover in *cerrado denso* is ∼60%. There is large variation in the canopy cover of both vegetation types, however, so there can be strong gradients in canopy cover in landscapes where they abut. At Panga Station, for instance, the canopy cover gradient in the *Cerrado ralo*/*Cerrado denso* mosaic ranges from 0–95% (Mean = 52% ± 33.1 SD; Fig. 1A).

Our focal ecosystem engineer is *Atta laevigata*, whose large nest mounds are formed by workers depositing excavated soil around the main entrance to the nest. *Atta laevigata* is the most common *Atta* species in both *cerrado ralo* and *cerrado denso* (Costa & Vieira-Neto, 2016); *A. sexdens* is also found at Panga Station, but primarily in closed-canopy forest. In 2010 we haphazardly selected 10 active *A. laevigata* nests in each vegetation type (surface area of the *N* = 20 nests: 7–37 m^2^, mean = 16.7 m^2^ ± 6.7 SD). We then established three 1 ×2 m plots around each nest—one on the center of the nest mound, one immediately adjacent to the mound, and one 10 m from the mound edge (Appendix A). In each plot, we recorded the identity and height of all woody plants ≤1.2 m tall as well as data on environmental conditions (described below). Although there are some abandoned nests in our site, we restrict our analyses to active colonies because the effects of time-since-abandonment on environmental variables is unknown.

### Environmental data

Litter biomass in each plot was measured by collecting all litter from a randomly selected half of each plot once during the 2010–2011 rainy season, drying it at 50 °C for 72 h, and weighing it with a microbalance. Similarly, we dried and weighed all grasses from a randomly selected half of each plot to estimate above-ground grass biomass. Canopy cover above each plot was estimated using photos analyzed with Adobe Photoshop (Adobe Systems Inc., San Jose, California, USA). In our analyses we used the average canopy cover in two photos taken during the same rainy season. Photos were taken with a Nikon Coolpix 950 from a height of 50 cm (modified from Engelbrecht & Herz (2001) in either the early morning (6h) or early evening (18h).

At the end of the 2011 dry season we estimated surface soil moisture content in plots by collecting a sample of the top 20 cm of soil from two points separated by 100 cm. These samples were bulked, weighed, dried at 50 °C for 96 h, then weighed again to estimate percent moisture content. As a proxy for soil compaction we used soil penetrability: we dropped a 1 m long × 5 mm diameter iron rod vertically from a height of 50 cm at three haphazardly selected points in each plot, then measured the depth to which the rod penetrated the soil at each point. We used the average of these values in our analyses; these data were recorded at the end of the 2010–2011 rainy season. Finally, at the end of the 2010–2011 rainy season we also counted all woody and herbaceous plants ≤ 120 cm tall in each plot and identified them with the help of local specialists and comparison with the collections of the UFU herbarium (HUFU). Of the 1,827 stems recorded 25% could only be identified to genus and morphospecies.

During the 2011 rainy season we selected *N* = 5 nests in *Cerrado ralo* and *N* = 5 nests in *Cerrado denso* nests for analyses of soil chemistry. For each nest we collected soils in the plots on nest mounds and the plots 10m from nests. We collected five soil samples of ∼100 g samples each from each plot: one from the plot center and one from each corner. The five samples from each plot were bulked into a single sample and taken to the Soils Analysis Lab of the Federal University of Uberlândia (UFU), where pH, P, K^+^, Ca^2+^, Mg ^2+^, Al^3+^, and total organic matter were measured using their standard protocols (EMBRAPA, 1997).

### Statistical analyses: does *Atta laevigata* modify the gradient in canopy cover?

To test for an effect of plot proximity to *A. laevigata* nests on logit-transformed canopy cover (Warton & Hui, 2011) we used using Generalized Linear Mixed Models (GLMMs Bolker et al., 2009). The significance of plot proximity was assessed by comparing the model including only the random effect of nest identity with models including this random effect, plot proximity to ant nests, nest mound area as a covariate, and plot location × covariate interactions. All models used a Gaussian distribution with an identity function; nest mound area was not included as a covariate because preliminary analyses indicated it did not improve the fit of models.

### Statistical analyses: do environmental conditions that influence plant establishment vary with canopy cover or proximity to *A. laevigata* nests?

We used Principal Components Analyses (PCA) to summarize environmental conditions in each plot because many of the biophysical variables we measured were highly correlated (Appendix B). The complete suite of environmental data was only collect in a subset of *N* = 10 nests, so we conducted two separate PCAs. The first was conducted using the environmental data collected in all *N* = 60 plots (i.e., plots on, adjacent to, and far from all *N* = 20 nests). These variables were: total grass biomass, total litter biomass, soil penetrability, surface soil moisture content, and percent canopy cover. The second was conducting using the subset of *N* = 10 nests for which we also collected data on soil chemistry; it was therefore the most comprehensive with respect to the environmental variables included, but was more limited in nest number and plot location because it only included plots on and far from the *N* = 10 nests. We hereafter refer to these PCAs as “PCA-1” and “PCA-2”, respectively. Both were conducted with correlation matrices because of the scales of each variable were different.

Each plot’s PCA scores are new variables that summarize local environmental conditions after controlling for correlation among the variables measured, and can therefore be used as dependent or independent variables in subsequent analyses (sensu Baiser et al., 2012). To determine if environmental conditions in a plot vary with canopy cover or proximity to *A. laevigata* nests we used a plot’s score on the 1st Principal Component Axis as the dependent variable in Generalized Linear Mixed Models with Gaussian errors. Plot location (i.e., on, adjacent to, or far from the nest) was a main effect with canopy cover included as a covariate. Although the effects of large colonies could potentially extend further from the nest boundary than those of smaller ones (Costa et al., 2008), we did not include nest area as a covariate because preliminary analysis indicated it did not improve the fit of models including just canopy cover. However, we did include nest identity as a random effect. The resulting models were ranked with Akaike Information Criteria (Burnham & Anderson, 2002) to determine which model best fit the observed data.

### Statistical analyses: do *A. laevigata* and canopy cover act independently or in concert to influence plant abundance and species richness?

We used two sets of Generalized Linear Mixed Models with Poisson error distributions to determine if plant abundance and species richness in plots were best explained by ant-related factors (proximity to leaf-cutter ant nests, environmental conditions in plots), canopy cover, or a combination of both. The two analyses that were identical except for the PCA scores used to summarize local environmental conditions: the first group of models used the axis scores from ‘PCA-1’ i.e., all nests and plots but no data on soils), while the second used the axis scores from ‘PCA-2’ all environmental variables but fewer nests and plot locations). Juvenile plant abundance or richness were the dependent variables. Once again preliminary analyses indicated including colony area did not improve the fit of models. Nest identity was again included as a random effect; because of significant overdispersion the models for plant abundance also included a random per-observation term.

All analyses were conducted using the R statistical programming language (R Core Development Team, 2014). For the GLMMs we used the lme4 package (Bates et al., 2015), while PCAs were conducted with package ggbiplot (Vu, 2015).

## Results

We surveyed *N* = 1257 individual plants from *N* = 66 genera. We were able to identify 89% of these stems, with the remainder assigned to one of *N* = 35 morphospecies (Appendix C). Most individuals were trees (62.6%) or shubs (26.6%), with only 10.8% of stems being woody vines. The most common species recorded were *Miconia albicans* (Melastomataceae, *N* = 239), *Tapirira guianensis* **(***Anacardiaceae* **,** *N* = 98), *Matayba guianensis* (Sapindaceae, *N* = 66), *Cordiera myrciifolia* (Rubiaceae, *N* = 65) and *Serjania erecta* (Sapindaceae, *N* = 57); these five species represent 42% of the stems sampled (Appendix C). Average plant height was 16.6 cm ± 20.9 SD; 83% of the stems were ≤30 cm tall and 56% were”- ≤10 cm tall, suggesting most were seedlings or relatively recent recruits.

### Does *Atta laevigata* modify the canopy cover gradient?

The model that best fit the data on the amount of canopy cover over a plot is the one including only the random effect of nest identity (Table 1). This indicates *A. laevigata* colonies alter canopy cover around their nests, but not in a systematic way related to nest size, and that there is no predictable change in canopy cover as a function of proximity to ant nests (Fig. 1B).

### Do environmental conditions that influence woody plant establishment vary with canopy cover or proximity to *Atta laevigata* nests?

**Figure 2:**
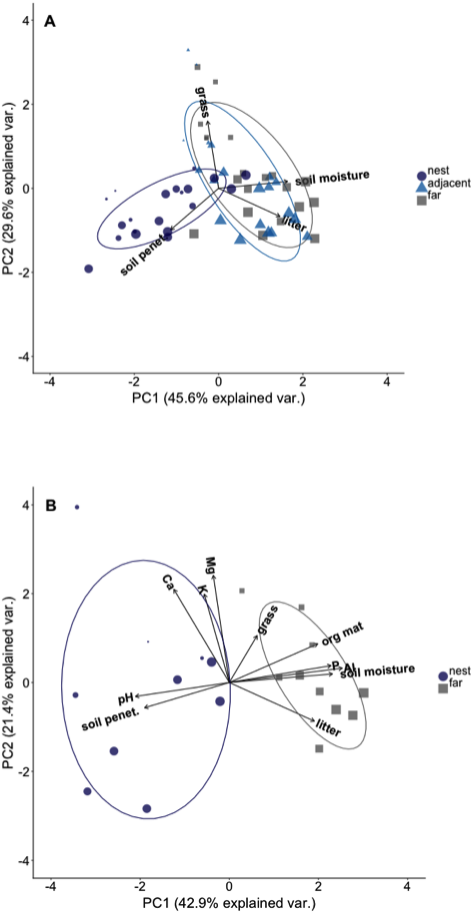
Principal component ordination of the environmental conditions in plots at different distances from *Atta laevigata* nests. (A) Ordination of the environmental conditions in plots located on (blue circles), adjacent to (blue triangles), or 10 m from the edge (gray squares) of *N* = 20 *Atta laevigata* nests. The variables included in this PCA (referred to as PCA-1 in the text) were litter biomass, soil penetrability, grass biomass, and soil moisture content. (B) ordination of the environmental conditions in plots located on (blue circles) or 10 m from (gray squares) the mounds of *N* = 10 *Atta laevigata* nests. This analysis, referred to as PCA-2 in the text, also included measurements of on soil pH, soil macronutrients, and soil organic material in plots. Symbol size in both figures indicates the percent canopy cover over that plot.

Plots on nest edges and those far from nests overlapped in ordination space, indicating they had very similar environmental conditions (Fig. 2A). However, there was almost no overlap in ordination space between either of these locations and the plots located in the middle of *A. laevigata* nest mounds (Fig. 2A), even when the number of nests was reduced to include soil data (Fig. 2B). In ‘PCA-1’ the first axis explained 45.6% of the variance and was positively correlated with litter biomass and soil moisture content. The second axis explained an additional 29.6% of the variance; it was negatively correlated with grass biomass and soil penetrability (Table 2). In ‘PCA-2’ the first axis explained 42.9% of the variance and was positively correlated with litter and grass biomass, soil moisture content, and soil P, Al^3+^, and organic material (Table 3). The second axis explained 21.4% of the variance and was positively correlated with all other environmental variables measured. In light of these results we used the scores from the first principal components as the dependent variable in subsequent analyses.

**Table 1:** Generalized Linear Mixed Model selection for the effect of plot proximity to leaf-cutter ant (*Atta laevigata*) nests on canopy cover in plots. The significance of plot proximity was assessed by comparing the model including only the random effect of nest identity (model 1) with models including this random effect, plot proximity to ant nests, and nest mound area as a covariate (model 2: no plot location × covariate interaction; model 3: main effects of plot location, the covariate, and a plot location × covariate interaction). All models used a Gaussian distribution with an identity function; nest mound area was not included as a covariate because preliminary analyses indicated it did not improve the fit of models. Considering the location of plots or nest mound area does not improve the fit to the data, indicating canopy cover is independent of proximity to ant nests and nest mound size. The best model is noted in bold.

Model	Factors	Resid. Df	Resid. Dev	*d*AIC	wAIC
**1**	**Nest Identity**	**57**	**97.525**	**0**	**0.999**
2	Nest Identity, Plot Location, Nest Mound Area	54	94.098	14.567	6.8 × 10^−4^
3	Nest Identity, Plot Location, Nest Mound Area, Plot Location*Nest Mound Area Interaction	52	90.144	29.186	4.6 × 10^−7^

**Table 2:** Factor loadings for the four principal components axes summarizing environmental variables measured in study plots located in Brazilian Cerrado. The cumulative proportion of the variance explained by these axes = 100%. The variables included in this PCA (referred to as PCA-1 in the text) were litter biomass, soil penetrability, grass biomass, and soil moisture content. Data for PCA-1 were collected in plots on the center of, adjacent to, and 10 m from the edge of *N* = 20 all *Atta laevigata* nest mounds.

Variable	PC1	PC2	PC3	PC4
Litter biomass	0.5864	−0.3345	0.4237	−0.6039
Soil penetrability	−4.4584	−0.4926	0.6753	0.3016
Grass biomass	−0.1053	0.7990	0.5749	−0.1415
Soil moisture content	0.6594	0.0855	0.1845	0.7241

**Table 3:** Factor loadings for the first four principal component axes summarizing environmental variables measured in study plots in Brazilian Cerrado. The summed proportion of the variance explained by these axes is 84.9%. The variables included in this PCA (referred to as PCA-2 in the text) were litter biomass, soil penetrability, grass biomass, soil moisture content, soil pH, several soil macronutrients, and soil organic material, and the data were collected in plots in the center of and 10 m from *N* = 10 *Atta laevigata* nest mounds.

Variable	PC1	PC2	PC3	PC4
Litter biomass	0.3227	−0.2081	0.3570	−0.1962
Soil penetrability	−0.3214	−0.1359	−0.2194	−0.4393
Grass biomass	0.1052	0.2517	−0.6646	−0.3390
pH	−0.3559	−0.0750	−0.1816	0.3599
P	0.3858	0.0906	0.2009	0.0639
K^+^	−0.0948	0.4755	0.2186	−0.5393
Ca^2+^	−0.2101	0.5013	0.0961	0.3533
Mg^2+^	−0.0607	0.5764	0.2763	0.1293
Al^3+^	0.4283	0.0770	0.0190	−0.0571
Organic material	0.3354	0.2080	−0.3874	0.0090
Soil moisture content	0.3914	0.0460	0.1600	0.2902

**Figure 3:**
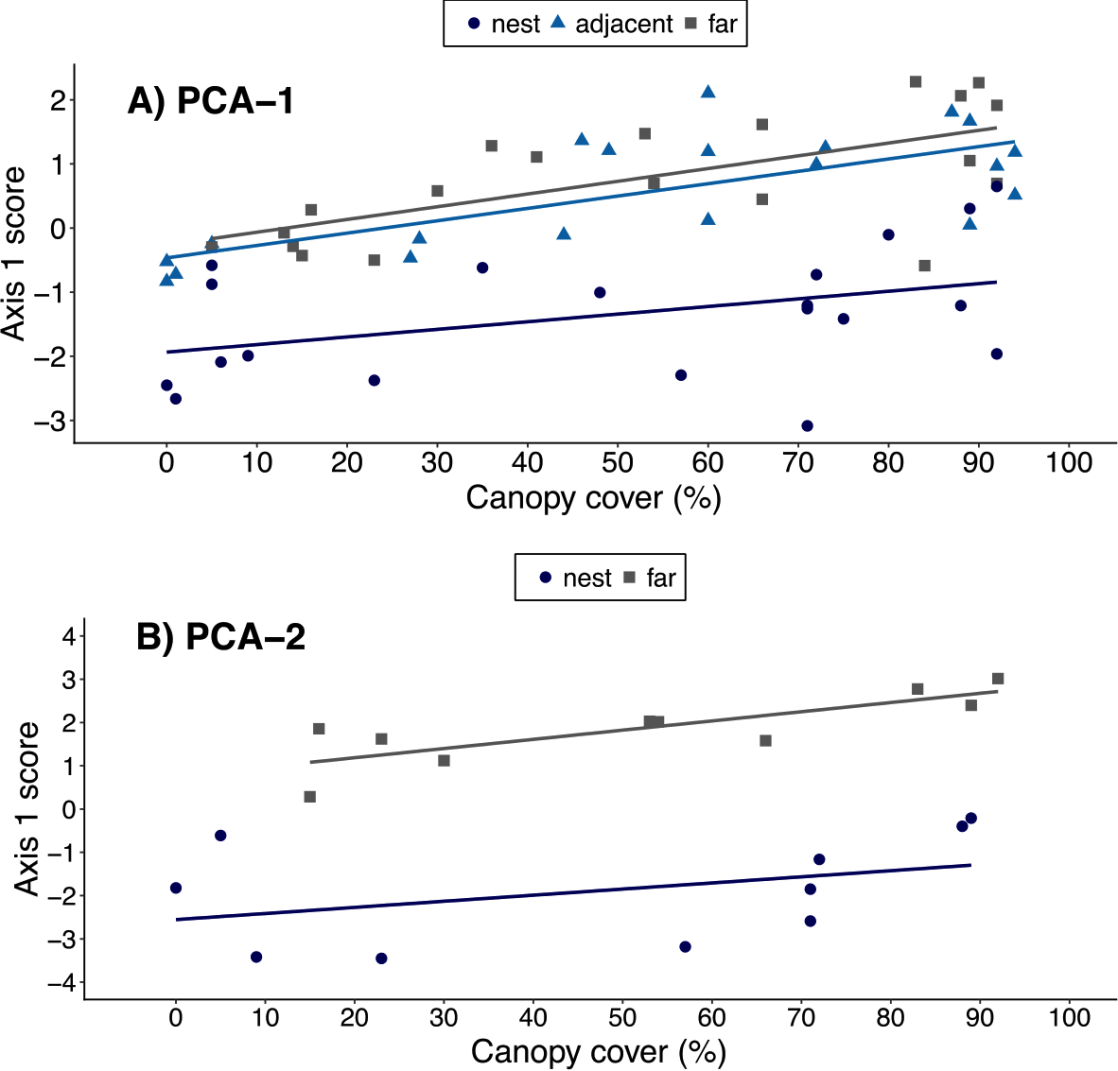
Relationship between canopy cover over a plot and environmental conditions in that plot. Environmental conditions were each plot’s score on the 1st principal component of either (A) PCA-1 (*N* = 5 environmental variables measured for *N* = 20 nests) or (B) PCA-2 (*N* = 12 environmental variables measured for *N* = 10 nests). Plots were on the middle of nest mounds (blue circles), adjacent to nests (blue triangles), or 10 m from the edge of nests (gray squares). The linear regression lines for each group of plots are shown in the corresponding colors.

When using the results of ‘PCA-1’, canopy cover over a plot was positively correlated with a plot’s PCA1 score (*ρ* = 0.44, *t* = 3.77, *df* = 58, *p* < 0.001), suggesting an association between canopy cover and local environmental conditions. However, the best-fit model included both canopy cover and plot location. This indicates leaf-cutter ants also influenced environmental conditions, but that the magnitude of the effect varied with plot proximity to nests (Table 4, Fig. 3A). When data on soils were included in the PCA, however, there was no longer a correlation between canopy cover over a plot and that plot’s score on the 1st PCA axis (*ρ* = 0.30, *t* = 1.35, *df* = 18, *p* = 0.20). Furthermore, the model that best fit the data on environmental conditions in a plot only included the proximity of a plots to ant nests and the random effect of nest identity (Table 5, Fig. 3B). In other words, the impact of ants on environmental conditions influencing establishment far outweighs that of canopy cover, but this is only revealed once data on soil properties are included in the analyses.

**Table 4:** Generalized Linear Mixed Model selection for the effect of plot proximity to leaf-cutter ant nests vs. canopy cover on environmental conditions in plots (based on PCA-1 scores for the 1st axis). The significance of these factors was assessed by comparing the models including only the random effect of nest identity (model 1) with models including this random effect and plot location (model 2), canopy cover (model 3), plot location and canopy cover (model 4), or nest identity, and plot location, canopy cover, and a plot location × canopy cover interaction (model 5). All models used a Gaussian distribution with an identity function; nest mound area was not included as a covariate because preliminary analyses indicated it did not improve the fit of models. The best fitting model (bold) was the one that included Plot Location, Canopy Cover, and the random effect of Nest Identity.

Model	Factors	Resid. Df	Resid. Dev	*d* AIC	wAIC
**4**	**Plot Location, Canopy Cover, Nest Identity**	**54**	**134.13**	**0**	**0.796**
2	Plot Location, Nest Identity	55	148.95	2.72	0.205
5	Plot Location, Canopy Cover, Plot Location*Canopy Cover Interaction, Nest Identity	52	132.09	19.88	3.8 × 10^−5^
3	Canopy Cover, Nest Identity	56	192.10	49.03	1.8 × 10^−11^
1	Nest Identity	57	205.28	51.12	6.2 × 10^−12^

**Table 5:** Generalized Linear Mixed Model selection for the effect of plot proximity to leaf-cutter ant nests vs. canopy cover on environmental conditions in plots (based on PCA-2 scores for the 1st axis). The significance of these factors was assessed by comparing the models including only the random effect of nest identity (model 1) with models including this random effect and plot location (model 2), canopy cover (model 3), nest identity and canopy cover (model 4), or nest identity, and plot location, canopy cover, and a plot location × canopy cover interaction (model 5). All models used a Gaussian distribution with an identity function. The best fitting model (bold) was the one that included the fixed effect of plot location and the random effect of nest identity.

Model	Factors	Resid. df	Resid. Dev	*d*AIC	wAIC
**2**	**Plot Location, Nest Identity**	**16**	**56.15**	**0**	**0.906**
4	Canopy Cover, Nest Identity	15	49.82	4.55	0.093
5	Plot Location, Canopy Cover, Plot Location*Canopy Cover Interaction, Nest Identity	14	49.47	13.75	9.4 × 10^−4^
1	Nest Identity	17	86.77	26.67	1.5 × 10^−6^
3	Canopy Cover, Nest Identity	16	84.86	33.41	5.0 × 10^−8^

### Do *A. laevigata* and canopy cover act independently or in concert to influence woody plant abundance and species richness?

We found 20.95 ± 18.14 SD recruits (range = 0–85) in each 2 m^2^ study plot. However, the mean number of recruits plot^−1^ decreased as one moved closer to the center of nests: plots far from nests had on average 29.55 ± 19.39 SD recruits in them vs. 24.9 ± 15.62 SD recruits plot^−1^ on nest margins and 8.4 ± 11.93 SD recruits plot^−1^ in the center of nest mounds. The mean number of species per plot was also lowest in plots on the center of nests (3.2 ± 2.9 SD) with almost four-fold higher species richness in plots on nest margins (10 ± 4.4 SD) and 10 m from nests (11.8 ± 4.6 SD).

**Figure 4:**
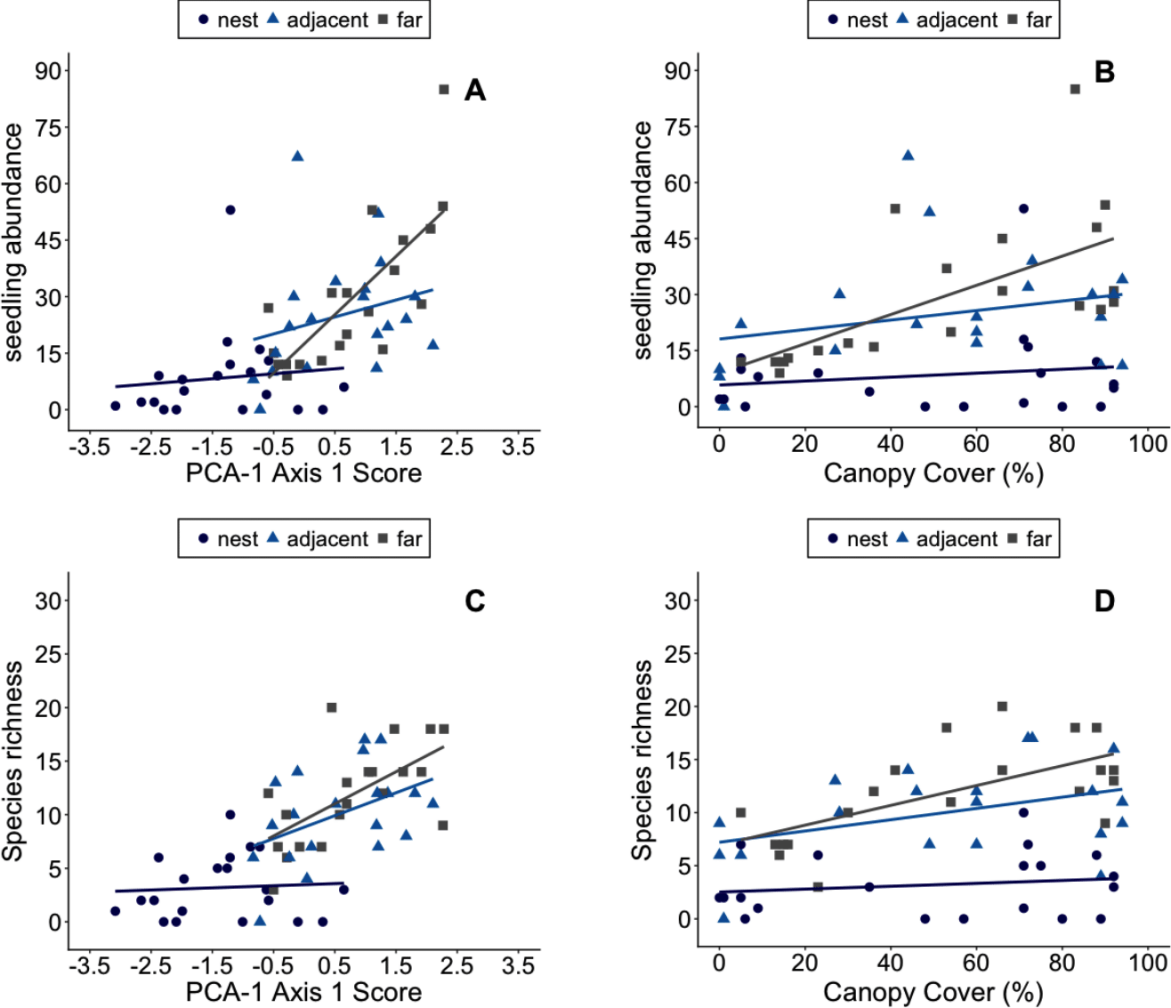
The number of plant recruits and species in plots with different environmental conditions. Environmental Conditions are defined as a plot’s score on the first principal component axis of PCA-1 (A–B). The number of plant recruits and species in plots with different amounts of canopy cover (C–D). Plots were in the center of *Atta laevigata* nest mounds (blue circles), adjacent to nests (blue triangles), or 10 m from the edge of nests (gray squares). The linear regression lines for each group of plots are shown in the corresponding colors.

Plant abundance in plots appears to be primarily influenced by ant-related factors (Figs. 4A, 4B). The best fit models include the proximity of plots to ant nests and environmental conditions in plots (Tables 6 and 7), though note *d* AIC values for the model including canopy cover was <2. Species richness in plots, however, appears to be shaped by both canopy cover and *Atta*-related effects (Figs. 4C, 4D). These results were consistent whether the PCA used to summarize environmental conditions in plots included data on soils or not (Tables 6 and 7). The significant effect of nest identity also indicates that some nests exert larger or smaller effects on local the abundance and diversity of recruits than others of similar size.

**Table 6:** Model selection for effects of canopy cover vs. nest mound area, plot location, and local environmental conditions (i.e., PCA-1 scores) on seedling abundance and species richness. The significance of these factors was assessed by comparing the models including only the random effect of nest identity and per-observation random effects (model 1) with models including these random effects and canopy cover (model 2), random effects and those related to ants and the environment (model 3), or random effects and both canopy-cover and variables related to ants (model 4). All models used a Poisson distribution with a logit link function. The best fitting model (bold) included factors and covariates related ants and their activity.

Model	Factors	Resid. df	Resid. Dev	*d*AIC	wAIC
**Plant Abundance** (*Environment*=*PCA-1 axis 1*)					
**3**	**Ant-related factors (plot location, local surface conditions) and random effects**	**54**	**30.25**	**0**	**0.517**
4	Ant-related effects (plot location, local surface conditions), canopy cover, and random effects	53	31.30	0.13	0.483
2	Canopy cover and random effects	56	25.81	31.48	7.54 × 10^−8^
1	Random effect of nest identity and per-observation random effects	57	23.42	35.74	8.9 × 10^−9^
**Species Richness** (*Environment*=*PCA-1 axis 1)*					
**4**	**Ant-related effects** (**plot location, local surface conditions), canopy cover, and random effects**	**53**	**82.13**	**0**	**1.0**
2	Canopy cover and random effects	56	40.69	50.70	9.79 × 10^−12^
1	Random effect of nest identity and per-observation random effects	57	32.14	54.98	1.15 × 10^−12^
3	Ant-related factors (plot location, local surface conditions) and random effects	54	30.25	139.21	5.90 × 10^−31^

**Table 7:** Model selection for effects of canopy cover vs. nest mound area, plot location, and local environmental conditions (i.e., PCA-2 scores) on seedling abundance and species richness. The significance of these factors was assessed by comparing the models including only the random effect of nest identity and per-observation random effects (model 1) with models including these random effects and canopy cover (model 2), random effects and those related to ants (model 3), or random effects and canopy-cover and ant-related variables, and local environmental conditions (axis 1 scores from PCA-2), which analyses indicated were influenced by both canopy cover and proximity to ant nests (model 4). All models used a Poisson distribution with a logit link function. The best fitting model (in bold) included factors and covariates related ants and their activity.

Model	Factors	Resid. df	Resid. Dev	*d*AIC	wAIC
**Plant Abundance** (*Environment*=*PCA-2 axis 1*)					
**3**	**Ant-related effects (plot location, local conditions) and random effects**	**15**	**9.94**	**0**	**0.699**
4	Ant-related factors (plot location, environmental conditions), canopy cover, and random effects	14	10.24	1.93	0.266
1	Random effects	17	7.32	6.84	0.023
2	Canopy Cover and random effects	16	8.22	8.13	0.012
**Species Richness***(Environment*=*PCA-2 axis 1)*					
**4**	**Ant-related factors (plot location, local conditions), canopy cover, and random effect**	**15**	**31.98**	**0**	**0.717**
3	Ant-related factors (plot location, local conditions), canopy cover, and random effect	16	28.57	1.86	0.283
2	Canopy cover and random effect	17	82.28	45.55	9.2 × 10^−11^
1	Random effect of nest identity	18	78.90	50.16	9.2 × 10^−12^

## Discussion

Both ecosystem engineers and environmental gradients are known to exert strong effects on biodiversity, but it is unknown if in general they act independently or in concert. This is because empirical studies simultaneously evaluating the relative influence of engineers and gradients remain rare (e.g., Badano & Marquet, 2009; Kleinhesselink, Magnoli & Cushman, 2014). We quantified the abundance and diversity of woody plant recruits at different distances from *A laevigata* nests found along a canopy cover gradient, as well as data on environmental conditions influencing plant establishment, growth, and survivorship (Fig. 2). Our results suggest that plant diversity in plots is shaped by both leaf-cutter ants and canopy cover. However, seedling abundance in plots is primarily driven by the ecosystem engineer, which both harvests plants and alters demographically relevant environmental conditions.

Leaf-cutter ants in our savanna site engineer the habitat in many of the same ways *Atta* species in lowland forests do—by transporting large amounts of soil to the surface, modifying soil chemistry (Meyer et al., 2013; Moutinho, Nepstad & Davidson, 2003), clearing the soil surface of plant material (reviewed in Farji-Brener & Illes, 2000; Leal, Wirth & Tabarelli, 2014), and stripping tree canopies of leaves (Leal, Wirth & Tabarelli, 2014). However, our spatially stratified sampling around nests also revealed that leaf-cutter ants do not modify canopy cover, even directly over nest mounds. This suggests that neither increased light penetration to the understory nor changes in abiotic conditions resulting from increased light are mechanisms by which *A. laevigata* indirectly modifies communities of juvenile plants in our site. This conclusion contrasts sharply with that of prior studies (Correa et al., 2010; Meyer et al., 2011), but most of these have been conducted in lowland forests where light limitation is often the principal factor limiting seedling recruitment and growth (Kitajima, 1994). The relatively shorter stature of Cerrado tress results in far greater penetration of light to the understory, even in physiognomies like *Cerrado denso* where canopy cover can exceed 90%.

Instead, it appears that *A. laevigata* colonies create what Farji-Brener & Illes (2000) refer to as ‘bottom-up’ gaps: patches of unique habitat resulting from *Atta*’s modifications of the understory and soil surface. We hypothesize that *A. laevigata* indirectly increases seed mortality due to desiccation (Salazar et al., 2012a) and granivory (Costa, Vasconcelos & Bruna, 2017) by reducing soil moisture content and clearing away litter (Appendix D). We also hypothesize it reduces the growth or survival of plants that become established on nest mounds by altering soil chemistry through bioturbation, by altering nutrient availability (but see Sternberg et al., 2007), or burying them under excavated soil (Costa, 2013). If so, *A. laevigata’s* reduction of juvenile plant abundance via environmental engineering of the Cerrado may rival its direct effects as a seed predator (Costa, Vasconcelos & Bruna, 2017; Ferreira, Bruna & Vasconcelos, 2011) and herbivore (Costa, Vasconcelos & Bruna, 2017; Vasconcelos & Cherrett, 1997).

It is notable that the impacts of *A. laevigata* on the abundance and diversity of plant recruits appears restricted primarily to the nest mound itself, which may limit the spatial extent of an individual colony’s impact. However, a salient feature of many engineers is that their localized impacts can often persist long-term (Hastings et al., 2007). Because *Atta* mounds remain long after a colony has died or migrated, their impact on the spatial distribution of soil nutrients (Sousa-Souto, Schoereder & Schaefer, 2007) or the spread of fire (Carvalho et al., 2012) be an example of such persistence. If so, then *A. laevigata*’s short- and long-term footprint on a landscape may be strongly influenced by historical changes in population size. Such demographically dependent effects of engineers may be particularly common where their activities have clearly delineated boundaries that scale with individual, colony, or population size (Hastings et al., 2007). We suggest *Atta*’s landscape-level impacts are best assessed with models linking the dynamics of engineer populations with those of the patches they create (e.g., Wright, William & Jones, 2004).

### Implications for Cerrado plant communities

In Paleotropical savannas, canopy cover and herbivores interact in complex ways to influence soil properties and vegetation dynamics (Holdo & Mack, 2014; Rugemalila, Anderson & Holdo, 2016). In contrast to these ecosystems, however, the density and diversity of large mammalian herbivores in the Cerrado is very low (Marinho-Filho, Rodrigues & Juarez, 2002). This has led many to conclude that plant population and communities in this biome are largely structured by edaphic factors (reviewed in Hoffmann & Moreira, 2002; Mistry, 1998; Ruggiero et al., 2002) and that the influence of herbivores is negligible (e.g., Gardner, 2006). Although the key role of physical factors in plant recruitment in the Cerrado is indisputable (Hoffmann, 1996; Hoffmann, 2000; Salazar et al., 2012a; Salazar et al., 2012b), studies evaluating the impacts of herbivores are rare (Ferreira, Bruna & Vasconcelos, 2011; Mundim et al., 2012), especially those simultaneously assessing the effects of herbivores and edaphic conditions (e.g., Klink, 1996). Our study supports Costa et al.’s (2008) hypothesis that plant-herbivore interactions can herbivores play a dominant role in Cerrado plant demography. Furthermore, our results provide compelling evidence that leaf-cutter ants do so both directly by harvesting seedlings and as ecosystem engineers modifying the conditions influencing plant recruitment, growth, and survival. As such, failing to consider the myriad impacts of these keystone species will undermine attempts to develop general theory for vegetation dynamics in this biome (e.g., Gardner, 2006), as well as conservation and restoration efforts.

### Future directions

How the spatio-temporal impacts of engineers are influenced by disturbance type, frequency, and intensity is conceptually critical (Crain & Bertness, 2006) but conspicuously understudied (Hastings et al., 2007). Fire is one of the most important forms of disturbance in savannas worldwide, where it can strongly influence seedling establishment (e.g., Iacona, Kirkman & Bruna, 2010), environmental gradients, and the foraging of leaf-cutter ants (Lopes & Vasconcelos, 2011). However, the density and abundance of *Atta* nests can also influence how fire spreads (Carvalho et al., 2012) and post-fire nutrient availability (Sousa-Souto, Schoereder & Schaefer, 2007). We suggest future studies in this system should focus on how *Atta*’s engineering of seedling communities is influenced by fire and fire-*Atta* feedbacks. Understanding these disturbance-engineer interactions is especially important given how deforestation, road creation, and other human activities lead to more frequent fires (Nepstad et al., 1999) and elevated *Atta* abundance (Cameron & Bayne, 2009; Vasconcelos et al., 2006; Vieira-Neto, Vasconcelos & Bruna, 2016). The ecological and economic footprint of these engineers is therefore likely to increase dramatically in coming decades in ways that remain underappreciated and poorly understood.

##  Supplemental Information

10.7717/peerj.5612/supp-1 Appendix A *Atta laevigata* nest mounds and the arrangement of sampling plots Leaf-cutter ant (*Atta laevigata*) nest mounds in (A)* cerrado ralo* and (B) *cerrado denso* vegetation types. (C) the location of sampling plots relative to nest mounds. All photos by A. Costa.

10.7717/peerj.5612/supp-2 Appendix B Correlations of biophysical properties measured in a Brazilian Cerrado site with each other and canopy cover

10.7717/peerj.5612/supp-3 Appendix C The abundance and identity of woody plant species recorded in our study

10.7717/peerj.5612/supp-4 Appendix D Scatterplots of grass biomass, litter biomass, soil penetrability (i.e., penetration distance), and surface soil moisture content along a canopy cover gradient in Brazilian *Cerrado* Regression lines represent plots located at different distances from *Atta laevigata* nest mounds (i.e., plots located in the center of the nest mound, adjacent of the nest, and 10 m from the nest).

## Additional Information and Declarations

### Data Availability

The following information was supplied regarding data availability: Data used in this manuscript are available at Dryad: doi: http://dx.doi.org/10.5061/dryad.vf1gq0q. Code used for data entry, manipulation, analysis, and generating figures can be found at https://github.com/embruna/Costa-Atta-Seedlings and at Zenodo: http://doi.org/10.5281/zenodo.1418482.
