# Supplementary figures and images for: Do an ecosystem engineer and environmental gradient act independently or in concert to shape juvenile plant communities? Tests with the leaf-cutter ant Atta laevigata in a Neotropical savanna
Source: PeerJ. 2018 Oct 9;6:e5612. doi: 10.7717/peerj.5612 (PMC6183508; doi:10.7717/peerj.5612)

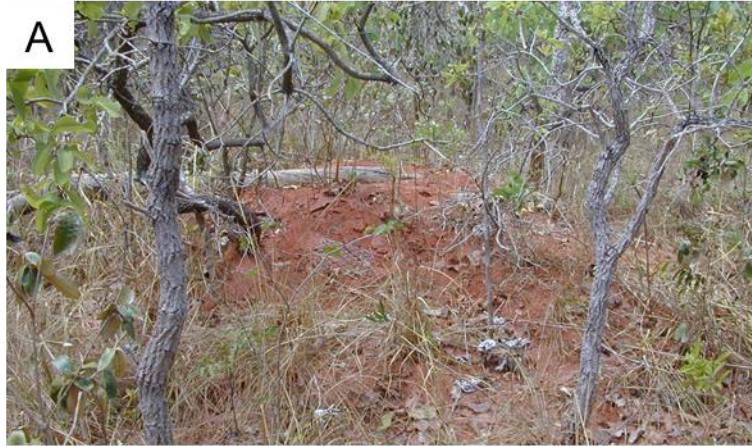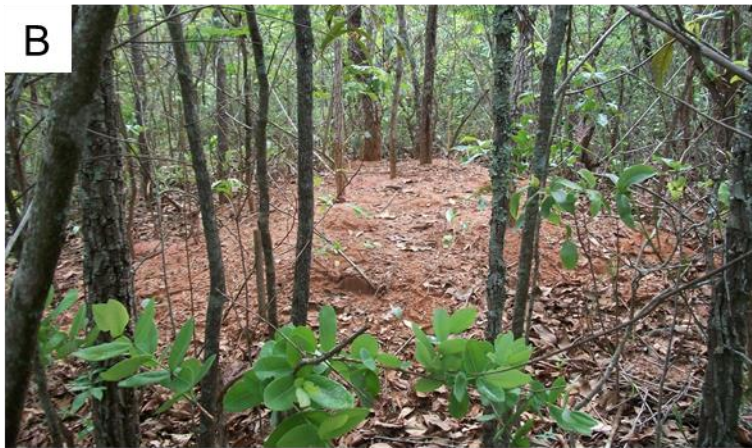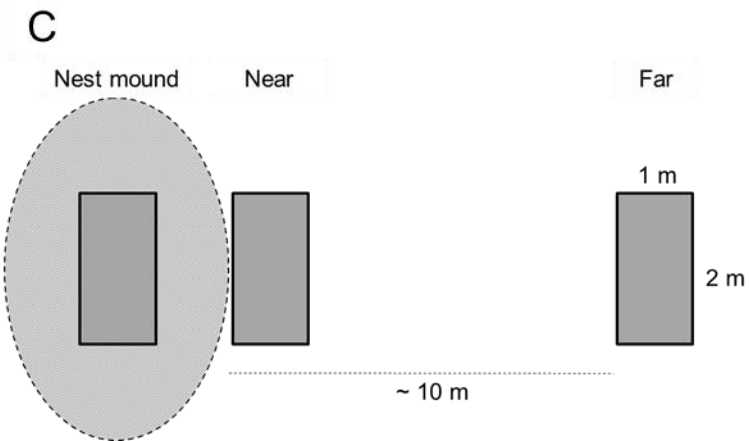

Supplement: Appendix A — Leaf-cutter ant (Atta laevigata) nest mounds in (A) cerrado ralo and (B) cerrado denso vegetation types. (C) the location of sampling plots relative to nest mounds. All photos by A. Costa. [file peerj-06-5612-s001.pdf]

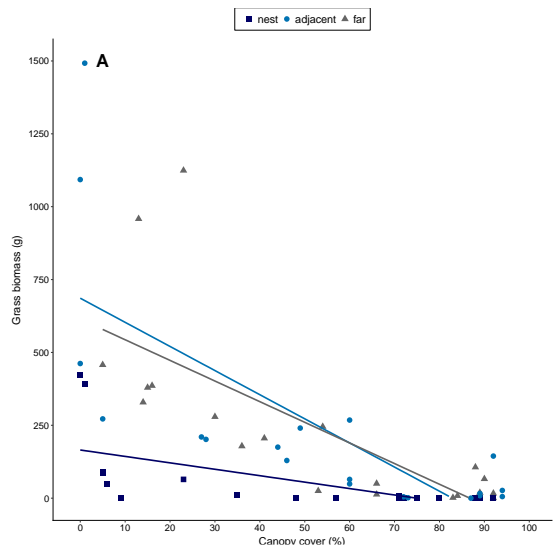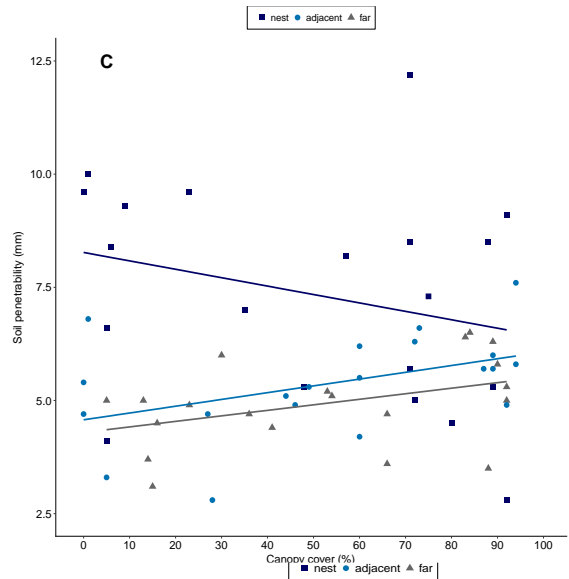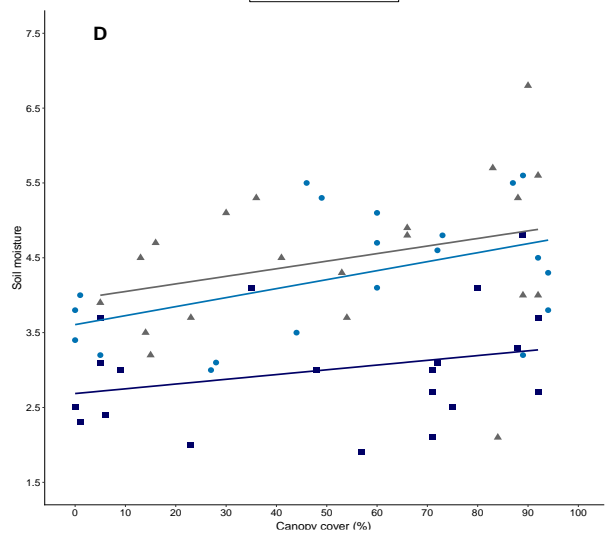

Supplement: Appendix D — Regression lines represent plots located at different distances from Atta laevigata nest mounds (i.e., plots located in the center of the nest mound, adjacent of the nest, and 10 m from the nest). [file peerj-06-5612-s004.pdf]
