# Supplementary material for: Do an ecosystem engineer and environmental gradient act independently or in concert to shape juvenile plant communities? Tests with the leaf-cutter ant Atta laevigata in a Neotropical savanna
Source: PeerJ. 2018 Oct 9;6:e5612. doi: 10.7717/peerj.5612 (PMC6183508; doi:10.7717/peerj.5612)
Supplement: Appendix B [file peerj-06-5612-s002.docx]

|  | Litter biomass | Soil penetrability | Grass biomass | pH | P | K | Ca | Mg | Al | Org. Material | Soil moisture  content |
| --- | --- | --- | --- | --- | --- | --- | --- | --- | --- | --- | --- |
| Canopy cover (%) | **0.5584** | 0.0070 | **-0.6734** | -0.3087 | 0.3396 | -0.4265 | **-0.5505** | -0.3775 | 0.1611 | -0.1829 | **0.3095** |
| Litter biomass | - | -0.0995 | -0.0524 | **-0.5070** | **0.6308** | -0.1796 | **-0.5274** | **-0.2458** | **0.6477** | **0.2411** | **0.6165** |
| Soil penetrability |  | - | 0.102 | 0.4222 | **-0.5584** | 0.1673 | 0.0828 | -0.0034 | -0.6115 | -0.5999 | **-0.4438** |
| Grass biomass |  |  | - | -0.1094 | 0.1032 | 0.1639 | 0.0234 | 0.0234 | 0.2511 | **0.5737** | -0.0137 |
| pH |  |  |  | - | **-0.6329** | **-0.1229** | 0.3483 | 0.0000 | **-0.7300** | **-0.4653** | **-0.6108** |
| P |  |  |  |  | - | -0.0492 | -0.2137 | 0.0705 | **0.7420** | 0.4995 | **0.7301** |
| K |  |  |  |  |  | - | **0.4850** | **0.6382** | -0.1128 | -0.0435 | -0.2032 |
| Ca |  |  |  |  |  |  | - | **0.7882** | -0.3531 | -0.1257 | -0.2392 |
| Mg |  |  |  |  |  |  |  | - | 0.0331 | 0.0494 | 0.0417 |
| Al |  |  |  |  |  |  |  |  | - | **0.7113** | **0.7521** |
| Org. material |  |  |  |  |  |  |  |  |  | - | **0.5788** |
| Soil moisture content |  |  |  |  |  |  |  |  |  |  | - |
