# Supplementary material for: Do an ecosystem engineer and environmental gradient act independently or in concert to shape juvenile plant communities? Tests with the leaf-cutter ant Atta laevigata in a Neotropical savanna
Source: PeerJ. 2018 Oct 9;6:e5612. doi: 10.7717/peerj.5612 (PMC6183508; doi:10.7717/peerj.5612)
Supplement: Appendix C [file peerj-06-5612-s003.docx]

| Species | Habit | No. of stems | (%) |
| --- | --- | --- | --- |
| *Miconia albicans* | tree | 239 | 19.01 |
| *Tapirira guianensis* | tree | 98 | 7.80 |
| *Matayba guianensis* | tree | 66 | 5.25 |
| *Cordiera myrciifolia* | shrub | 65 | 5.17 |
| *Serjania erecta* | vine | 57 | 4.53 |
| *Banisteriopsis anisandra* | vine | 30 | 2.39 |
| *Virola sebifera* | tree | 29 | 2.31 |
| *Guapira graciliflora* | tree | 26 | 2.07 |
| *Morphospecies* 24 | shrub | 26 | 2.07 |
| *Morphospecies* 93 | tree | 25 | 1.99 |
| *Qualea grandiflora* | tree | 25 | 1.99 |
| *Banisteriopsis stellaris* | vine | 24 | 1.91 |
| *Cardiopetalum calophyllum* | tree | 23 | 1.83 |
| *Myrcia splendens* | tree | 22 | 1.75 |
| *Smilax brasiliensis* | vine | 22 | 1.75 |
| *Eugenia involucrata* | shrub | 21 | 1.67 |
| *Solanum lycocarpum* | tree | 21 | 1.67 |
| *Cordiera obtusa* | shrub | 18 | 1.43 |
| *Roupala montana* | tree | 18 | 1.43 |
| *Casearia sylvestris* | shrub | 17 | 1.35 |
| *Maprounea guianensis* | tree | 16 | 1.27 |
| *Tabebuia* sp. | tree | 15 | 1.19 |
| *Brosimum gaudichaudii* | tree | 13 | 1.03 |
| *Coussarea hydrangeifolia* | tree | 12 | 0.95 |
| *Securidaca rivinifolia* | shrub | 12 | 0.95 |
| Morphospecies 65 | shrub | 11 | 0.88 |
| *Rourea induta* | shrub | 11 | 0.88 |
| *Myrcia variabilis* | tree | 10 | 0.80 |
| Morphospecies 56 | shrub | 9 | 0.72 |
| Morphospecies 62 | shrub | 9 | 0.72 |
| Morphospecies 74 | shrub | 9 | 0.72 |
| *Qualea parviflora* | tree | 9 | 0.72 |
| *Tachigali vulgaris* | tree | 9 | 0.72 |
| *Anacardium humile* | shrub | 8 | 0.64 |
| *Erythroxylum deciduum* | shrub | 8 | 0.64 |
| Morphospecies 31 | shrub | 8 | 0.64 |
| *Neea theifera* | shrub | 8 | 0.64 |
| *Protium heptaphyllum* | shrub | 8 | 0.64 |
| *Siparuna guianensis* | tree | 8 | 0.64 |
| *Styrax* sp. | tree | 8 | 0.64 |
| *Andira vermifuga* | tree | 7 | 0.56 |
| *Miconia fallax* | shrub | 7 | 0.56 |
| Morphospecies 64 | shrub | 7 | 0.56 |
| *Myrcia tomentosa* | tree | 7 | 0.56 |
| *Rudgea viburnoides* | tree | 7 | 0.56 |
| *Xylopia aromatica* | tree | 7 | 0.56 |
| *Manihot* sp. | shrub | 6 | 0.48 |
| *Qualea multiflora* | tree | 6 | 0.48 |
| *Tocoyena formosa* | tree | 5 | 0.40 |
| *Annona tomentosa* | tree | 4 | 0.32 |
| Morphospecies 49 | shrub | 4 | 0.32 |
| *Myrcia guianensis* | tree | 4 | 0.32 |
| *Palicourea rigida* | shrub | 4 | 0.32 |
| *Symplocos pubescens* | tree | 4 | 0.32 |
| *Annona dioica* | tree | 3 | 0.24 |
| *Cordiera sessilis* | tree | 3 | 0.24 |
| *Dalbergia miscolobium* | tree | 3 | 0.24 |
| *Davilla elliptica* | shrub | 3 | 0.24 |
| *Guapira noxia* | tree | 3 | 0.24 |
| *Himatanthus obovatus* | shrub | 3 | 0.24 |
| Morphospecies 14 | shrub | 3 | 0.24 |
| Morphospecies 8 | shrub | 3 | 0.24 |
| *Ouratea nana* | shrub | 3 | 0.24 |
| *Aristolochia esperanzae* | vine | 2 | 0.16 |
| *Bredemeyera floribunda* | shrub | 2 | 0.16 |
| *Connarus suberosus* | shrub | 2 | 0.16 |
| *Dimorphandra mollis* | tree | 2 | 0.16 |
| *Erythroxylum suberosum* | shrub | 2 | 0.16 |
| *Erythroxylum tortuosum* | shrub | 2 | 0.16 |
| *Eugenia* sp. 2 | shrub | 2 | 0.16 |
| *Fridericia platyphylla* | shrub | 2 | 0.16 |
| *Heteropterys campestris* | shrub | 2 | 0.16 |
| *Hirtella glandulosa* | tree | 2 | 0.16 |
| *Leptolobium dasycarpum* | tree | 2 | 0.16 |
| Morphospecies 28 | shrub | 2 | 0.16 |
| Morphospecies 40 | shrub | 2 | 0.16 |
| Morphospecies 63 | shrub | 2 | 0.16 |
| *Ocotea corymbosa* | tree | 2 | 0.16 |
| *Ocotea pulchella* | tree | 2 | 0.16 |
| *Ouratea castaneifolia* | tree | 2 | 0.16 |
| *Pera glabrata* | tree | 2 | 0.16 |
| *Pouteria ramiflora* | tree | 2 | 0.16 |
| *Vatairea macrocarpa* | tree | 2 | 0.16 |
| *Aspidosperma macrocarpon* | tree | 1 | 0.08 |
| *Aspidosperma tomentosum* | tree | 1 | 0.08 |
| *Bauhinia rufa* | tree | 1 | 0.08 |
| *Bowdichia virgilioides* | tree | 1 | 0.08 |
| *Byrsonima intermedia* | shrub | 1 | 0.08 |
| *Eugenia sp1* | shrub | 1 | 0.08 |
| *Gochnatia polymorpha* | shrub | 1 | 0.08 |
| *Leptolobium elegans* | tree | 1 | 0.08 |
| Morphospecies 11 | shrub | 1 | 0.08 |
| Morphospecies 17 | tree | 1 | 0.08 |
| Morphospecies 18 | shrub | 1 | 0.08 |
| Morphospecies 22 | shrub | 1 | 0.08 |
| Morphospecies 23 | shrub | 1 | 0.08 |
| Morphospecies 38 | shrub | 1 | 0.08 |
| Morphospecies 46 | shrub | 1 | 0.08 |
| Morphospecies 47 | shrub | 1 | 0.08 |
| Morphospecies 5 | shrub | 1 | 0.08 |
| Morphospecies 57 | shrub | 1 | 0.08 |
| Morphospecies 59 | shrub | 1 | 0.08 |
| Morphospecies 66 | shrub | 1 | 0.08 |
| Morphospecies 75 | shrub | 1 | 0.08 |
| Morphospecies 76 | shrub | 1 | 0.08 |
| Morphospecies 77 | shrub | 1 | 0.08 |
| Morphospecies 78 | shrub | 1 | 0.08 |
| Morphospecies 81 | shrub | 1 | 0.08 |
| Morphospecies 85 | shrub | 1 | 0.08 |
| Morphospecies 86 | shrub | 1 | 0.08 |
| Morphospecies 88 | shrub | 1 | 0.08 |
| Morphospecies 94 | shrub | 1 | 0.08 |
| *Myrsine guianensis* | tree | 1 | 0.08 |
| *Ouratea hexasperma* | tree | 1 | 0.08 |
| *Plathymenia reticulata* | tree | 1 | 0.08 |
| *Plenckia populnea* | tree | 1 | 0.08 |
| *Stryphnodendron polyphyllum* | tree | 1 | 0.08 |
| *Styrax ferrugineus* | tree | 1 | 0.08 |
| *Vernonia* sp | vine | 1 | 0.08 |
| *Vochysia tucanorum* | tree | 1 | 0.08 |
| *Zeyheria montana* | tree | 1 | 0.08 |
